# Supplementary material for: Activity in human dorsal raphe nucleus signals changes in behavioural policy
Source: Nat Commun. 2026 Feb 14;17:1665. doi: 10.1038/s41467-026-68349-9 (PMC12909899; doi:10.1038/s41467-026-68349-9)
Supplement: Supplementary file 1 — Supplementary Information [file 41467_2026_68349_MOESM1_ESM.pdf]

## SUPPLEMENTARY METHODS

In the supplementary figures, we report several analyses of fMRI timecourse data. These analyses were conducted using the procedure described in the Methods section of the main text. The formulas for the GLMs are detailed below.

In GLMs S5.1 and S5.2, we tested the effect of policy switches on DRN activity in moving 6-trial windows:

**GLM S5.1**

BOLD = $\beta_0$constant + $\beta_1$congruent-switch-poor + $\beta_3$trial-number

**GLM S5.2**

BOLD = $\beta_0$constant + + $\beta_1$congruent-switch-rich + $\beta_3$trial-number

Per above, GLMS5.1 and S5.2 were run iteratively on moving 6-trial windows aligned to different points within a block of trials. The 6-trial window starting points ranged from trial-in-block=1 to trial-in-block=10.

In GLM S5.3, we tested whether trials following policy-switches were associated with changes in DRN activity:

**GLM S5.3**

BOLD = $\beta_0$constant + $\beta_1$trial-after-policy-switch + $\beta_2$trial-number

Where *trial-after-policy-switch* is a binary variable indicating trials that immediately followed policy-switch trials.

In GLM S5.4, we tested whether option-specific encounters following policy-switches were associated with changes in DRN activity:

**GLM S5.4**

BOLD = $\beta_0$constant + $\beta_1$encounter-after-policy-switch + $\beta_2$trial-number

Where *encounter-after-policy-switch* is a binary variable indicating trials where the previous encounter with the option available elicited a policy-switch.

In GLM S6.1, we tested whether activity in MBD represented the pursue-vs-reject decision made on each trial:

**GLM S6.1**

BOLD = $\beta_0$constant + $\beta_1$pursue-vs-reject + $\beta_2$trial-number

Where *pursue-vs-reject* is a binary variable indicating the pursue-vs-reject decision made on each trial.

In GLM S6.2, we tested whether activity in MBD represented a prediction-error-like quantity reflecting the difference between the currently available reward option, and the average of recently encountered options:

**GLM S6.2**

BOLD = $\beta_0$constant + $\beta_1$value-difference + $\beta_2$trial-number

Where *value-difference* is a continuous variable reflecting the difference between the reward-magnitude of the option available on trial *t*, and the average value of options on the preceding five trials (see Methods in main text).

In GLM S7.1, we tested whether subcortical ROIs (DRN, MBD, Hb, LC, MS) represented exploratory policy-switches that were incongruent with the environment:

**GLM S7.1**

BOLD = $\beta_0$constant + $\beta_1$incongruent-switch + $\beta_2$congruent-switch + $\beta_3$trial-number

Where *incongruent-switch* is a binary variable reflecting policy switches that are incongruent with respect to the environment (i.e. pursue-switch in rich environments, reject-switch in poor environments), and *congruent-switch* is a binary variable reflecting policy switches that are congruent with respect to the environment (i.e. pursue-switch in poor environments and reject-switch in rich environments).

Finally, in GLM S7.2, we compared activity in MS and DRN during incongruent switches, and congruent switches:

**GLM S7.1**

BOLD = $\beta_0$constant + $\beta_1$incongruent-switch + $\beta_2$congruent-rich-vs-congruent-poor + $\beta_3$trial-number

Where *incongruent-switch* is a binary variable described above, and *congruent-rich-vs-congruent-poor* contrasted activity during congruent switches in rich and poor environments, respectively (congruent-rich= –1; congruent-poor=1).

## SUPPLEMENTARY FIGURES

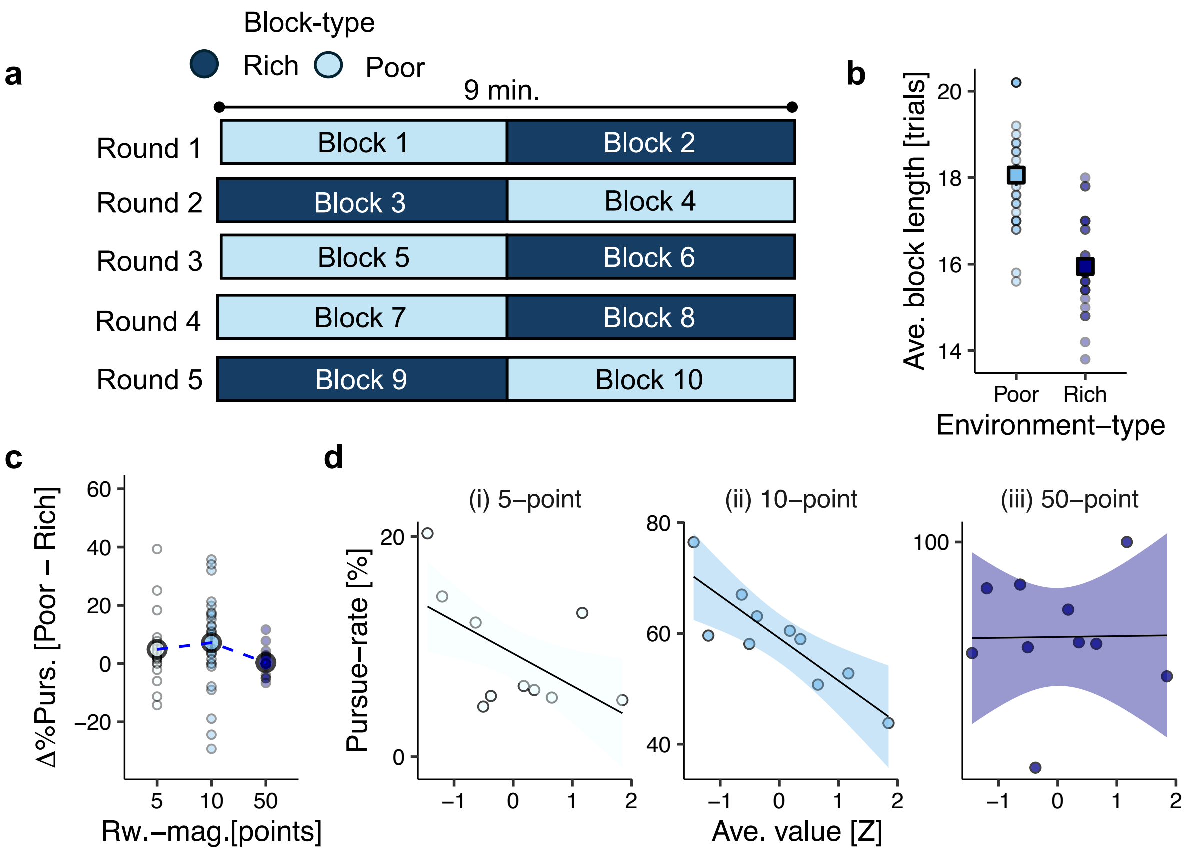


**Figure S1. Further details of behavioural task. (a)** Diagram round and block structure in an example session of the experiment. **(b)** Average number of trials completed in poor and rich blocks. Participants completed more trials in poor blocks because they pursued fewer opportunities in poor ($M$=44.20 ± 2.45%) compared to rich (M=69.00, ± 2.14%) blocks ($t_{poor\text{-}vs\text{-}rich}(26) = 15.52$, $p$ <.001). Dots show participant-level mean number of trials completed. **(c)** Change in pursue-rate for each reward-option between environments. A GLM predicting the probability of reward-pursuit indicated that, in addition to main effects of reward-magnitude ($\beta_{reward\text{-}magnitude}$= 6.11, SE = 0.21, $p$ < .001) and environment-type ($\beta_{environment\text{-}type}$= -2.01, SE = 0.14, $p$ < .001) there was a two-way interaction between these predictors ($\beta_{reward\text{-}magnitude*environment\text{-}type}$= -3.06, SE = 0.22, $p$ < .001) whereby participants pursued 10-point ($\beta_{environment\text{-}type}$ = -0.20, SE = 0.09, $p$ = .038) options more frequently in poor compared to rich environments, but did not show equivalent changes for 5-point ($\beta_{environment\text{-}type}$ = -0.51, SE = 0.28, $p$ = .064) or 50-point ($\beta_{environment\text{-}type}$ = -0.11, SE = 0.24, $p$ = .655) options. Dots indicate participant-level difference-scores [$\Delta = \mu(\text{pursue})_{poor} - \mu(\text{pursue})_{rich}$]. **(d)** Relationship between pursue-rate and average value (see Methods). A GLM predicting probability of reward-pursuit as a function of the average-value indicated participants were more likely to pursue 10-point ($\beta_{average\text{-}value}$ = -0.33, SE = 0.11, $p$ = .003) and 5-point ($\beta_{average\text{-}value}$ = -0.40, SE = 0.18, $p$ = .021) options as average-value decreased. There was no evidence for this effect for 50-point opportunities ($\beta_{average\text{-}value}$ = -0.14, SE = 0.47, $p$ = .765). Dots and error bars indicate mean ± SEM pursue-rate in deciles of average-value; line and shading indicate regression line ± SE. In all panels, n=27.

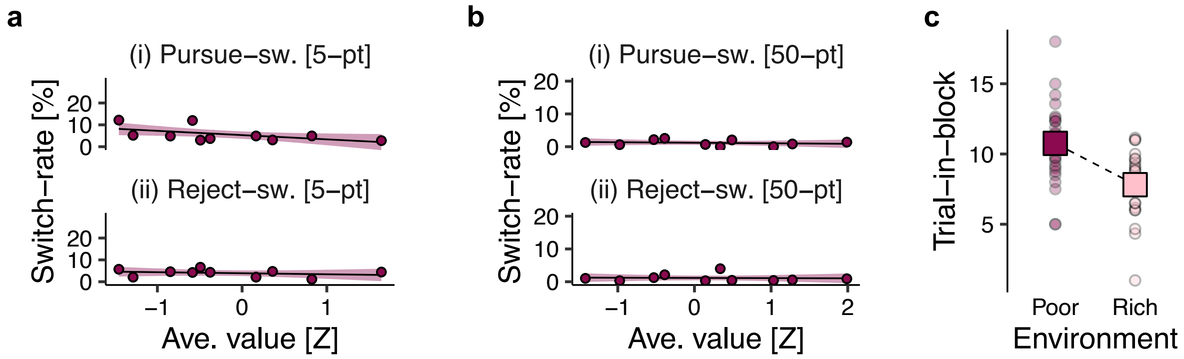


**Figure S2. Further detail on policy-switches. (a)** Relationship between average-value and probability of policy switches for 5-point option. A GLM predicting policy-switch as a function of average value provided no evidence that policy-switches for 5-point options were modulated by average value ( $\beta_{pursue\text{-}switch}$ = -0.26, SE = 0.14, $p$ =.071; $\beta_{reject\text{-}switch}$= -0.03, SE = 0.47, $p$ = .873). Dots and error bars indicate mean ± SEM switch-rate in deciles of average-value; line and shading indicates regression-line ± SE. **(b)** Relationship between average-value and probability of policy switches for 50-point option. A GLM predicting policy-switch as a function of average value provided no evidence that policy-switches for 50-point options were modulated by average value ($\beta_{pursue\text{-}switch}$ = -1.36, SE = 0.84, $p$ =.107; $\beta_{reject\text{-}switch}$= -0.12, SE = 0.24, $p$ = .606). Dots and error bars indicate mean ± SEM switch-rate in deciles of average-value; line and shading indicates regression-line ± SE. **(c)** Mean time (trial-in-block) at which congruent policy-switches occurred. Note that vast majority of switches occur at or after the midpoint of blocks, suggesting that participants accumulated evidence for an environment change before altering their behaviour. Further analysis indicated that congruent policy-switches in rich blocks (*M*=7.81, *SD* = 2.51) occurred earlier than congruent policy-switches in poor blocks (*M*=10.8, *SD*=2.94), and this difference was statistically significant ($\beta_{rich\text{-}vs\text{-}poor}$=-0.23, SE=0.06, $p$<.001). Squares indicate sample-level mean timepoints; dots indicate participant-level mean timepoints. In all panels, n=27.

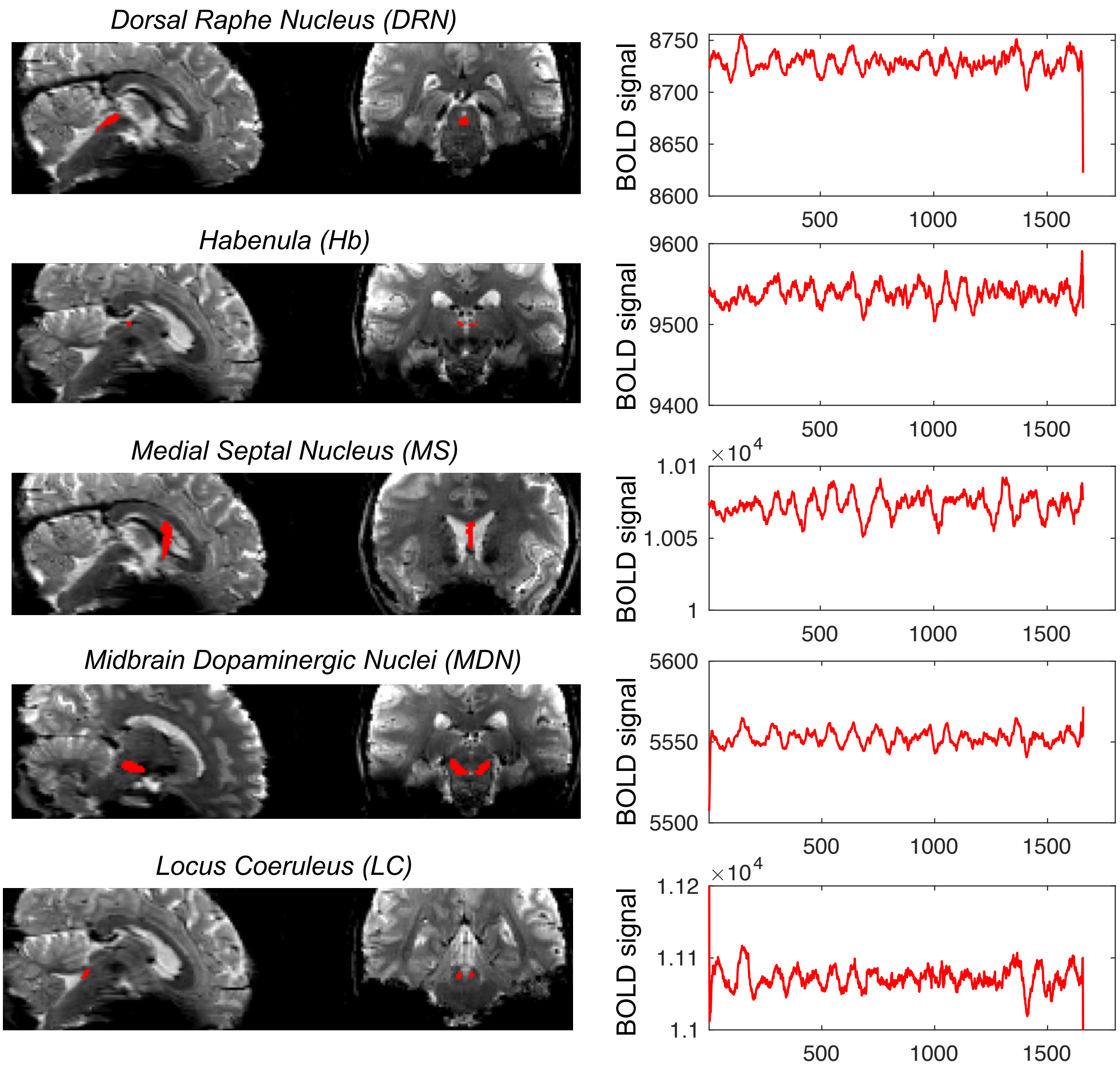


**Figure S3. Details of anatomical masks for subcortical regions of interest.** Anatomical masks from an example session in functional space. Anatomical masks were designed for each ROI in the standard space (see fig.3A) and were transformed from the standard space to each subject's functional space by applying a standard-to-structural-to-functional warp. The masks are overlaid on functional image from a representative session. The extracted BOLD signal from each mask is displayed next to its corresponding ROI. The lines show the BOLD signal extracted and averaged from each voxel within the ROI, for the whole duration of the scanning session.

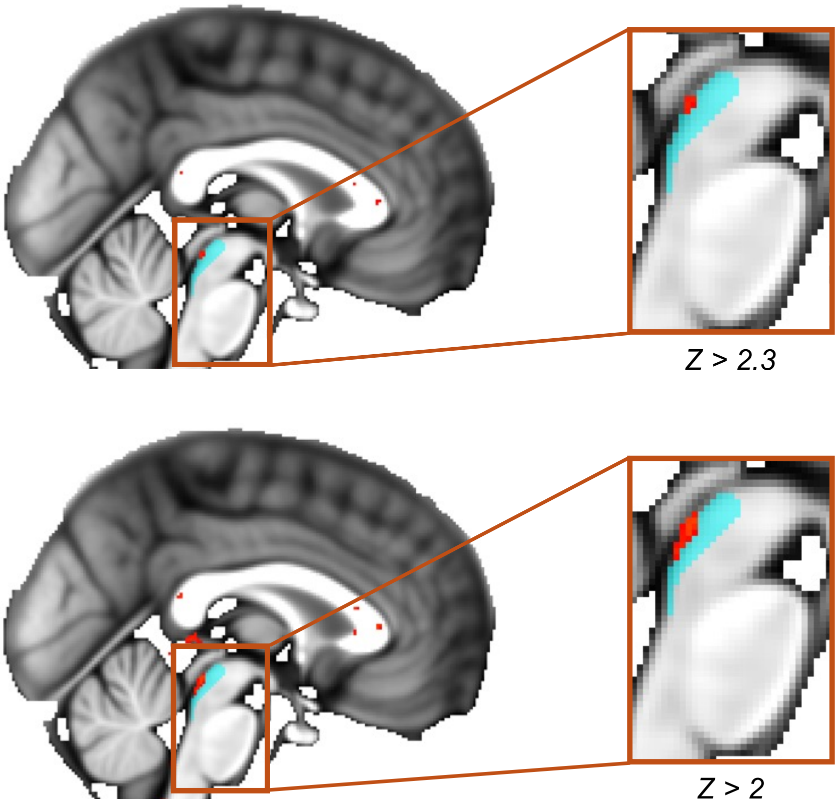


**Figure S4. Effect of policy-switch in the whole-brain.** We further examined the relationship between policy-switches and brain activity using a GLM with the same predictors as GLM4.1 in the main manuscript but implemented at the whole-brain level (GLM3.1). For the policy-switch effect no clusters in the brainstem survived correction. However, at a threshold of Z > 2.3 (approximately p = 0.01), we observed activity centred within our dorsal raphe nucleus (DRN) mask (shown in blue). Importantly, at this threshold, there were no statistically significant clusters of activity elsewhere in the brain, and nor did the DRN-related cluster reflect noise or spillover from the ventricles or adjacent regions. Furthermore, lower the threshold to Z = 2 indicated that the cluster expanded in a manner that was consistent with DRN's anatomical boundaries.

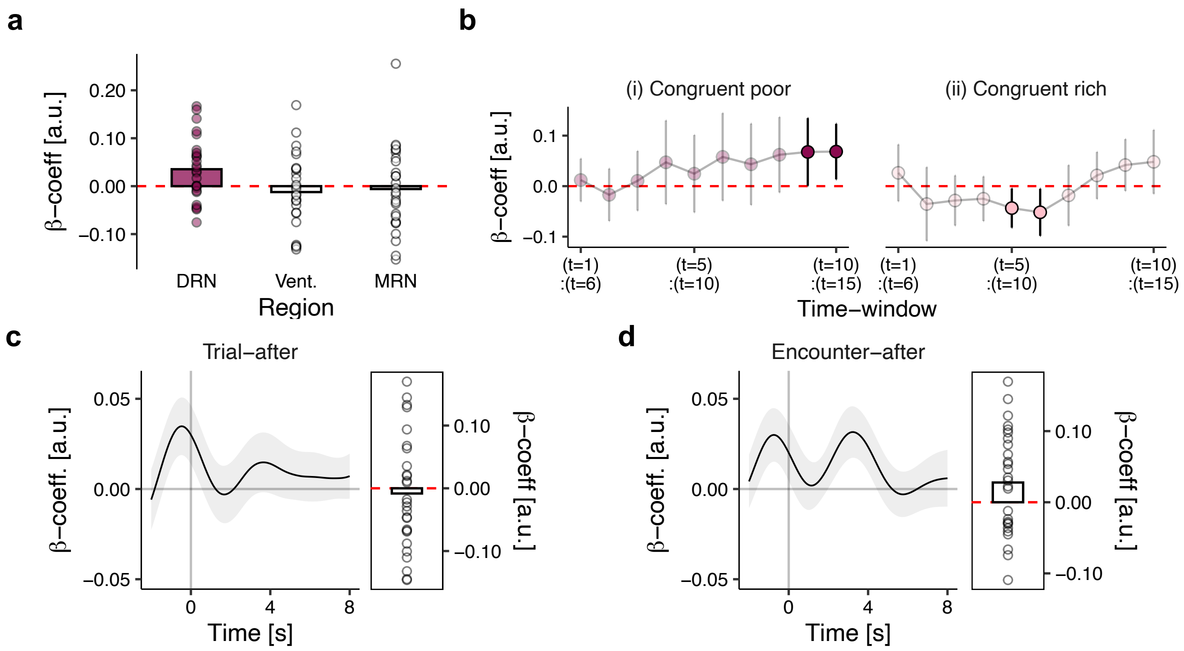


**Figure S5. Further details of policy-switch effect in DRN. (a)** Peak regression weights for policy-switch effect in dorsal raphe nucleus (DRN) and adjacent anatomical features including the 4th ventricle, and median raphe nucleus (MRN). There was no evidence that policy-switches affected BOLD signal in these areas (GLM4.1a; $t_{DRN}(26)=2.85$, $p$=.009, $t_{MRN}(26)$=-0.36, $p$=.724, $t_{Vent.}(26)$=-0.83, $p$=.415). Moreover, the policy-switch effect in DRN was greater than the effects in MRN and the 4th ventricle ($t_{DRN\text{-}vs\text{-}MRN}(26)$=2.08, $p$=.047, $t_{DRN\text{-}vs\text{-}Vent}(26)$=2.31, $p$=.028). **(b)** Effect of policy-switches on DRN activity in moving time windows within each block. Time window widths were 6 trials because this was approximately the SD of policy-switch time distributions in both rich blocks and poor blocks (see supplementary fig. S2C). Note that policy-switch effects only emerge in late time windows – i.e., (t=5):(t=10) and (t=6):(t=11) time windows in rich blocks, and (t=9:t=14) and (t=10);(t=15) time windows in poor blocks. Points and error-bars indicate mean ± 95%CI effect-size after Bonferroni correction. **(c)** Effect of the trial immediately following a policy-shift (i.e., regardless of which option featured on that trial) and DRN activity. There was no evidence that DRN activity changed on trials after policy switches (GLM S5.1; $t_{trial\text{-}after}(26)$ = -0.47, p=.999), suggesting that DRN signals policy switches with transient patterns of activity. **(d)** Effect of the encounter after a policy-switch (i.e., the option-specific subsequent trial after a policy shift) on DRN activity. There was no evidence that DRN activity changed on encounters after policy switches GLM S5.1; $t_{trial\text{-}after}(26)$ = -0.47, p=.999), providing further evidence that DRN signals policy switches with transient, rather than sustained, patterns of activity (GLM S5.2; $t_{encounter\text{-}after}(26)$ = 1.99, p=.284). In (b, d), bars show sample-level mean peak regression weight; dots indicate participant-level peak regression weights. In all panels, n=27.

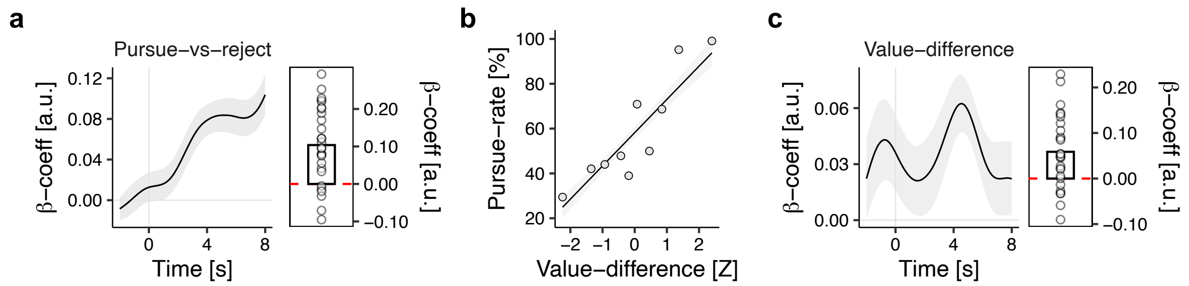


**Figure S6. Activity in midbrain dopaminergic nuclei. (a)** The pursue-vs-reject decision made on each trial modulated MBD activity (GLM S6.1; $t_{MBD;\ pursue\text{-}vs\text{-}reject}$ (26)=5.21, *p*<.001). Lines and shadings indicate mean ± SE of regression-weight across participants. Bar indicates sample-level mean peak regression weight; dots indicate participant-level peak regression weights. **(b)** Relationship between MBD activity and value-difference. **(b)** Participants were more likely to pursue reward-option as a function of value-difference – i.e., the difference between the reward magnitude afforded by an option and the average magnitude of recently encountered options ($\text{value-difference}_t$ = $\text{reward-magnitude}_t$ – $\text{average-value}_t$; $\beta_{\text{value-difference}}$= 0.90, SE = 0.07, *p* <.001). Dots and error bars indicate mean ± SEM pursue-rate in deciles of value-difference; line and shading indicate regression ± SE. **(c)** Activity in MBD was modulated by value-difference, which is reminiscent of a reward prediction error signal in the sense that average-value constitutes a simple prediction for the prospective reward-value of future trials (GLMS6.2; $t_{MBD;\ value\text{-}difference}$(26)=3.79, *p*<.001). Lines and shadings indicate mean ± SE of regression-weight across participants. Bar indicates sample-level mean peak regression weight; dots indicate participant-level peak regression weights. In all panels, n=27.

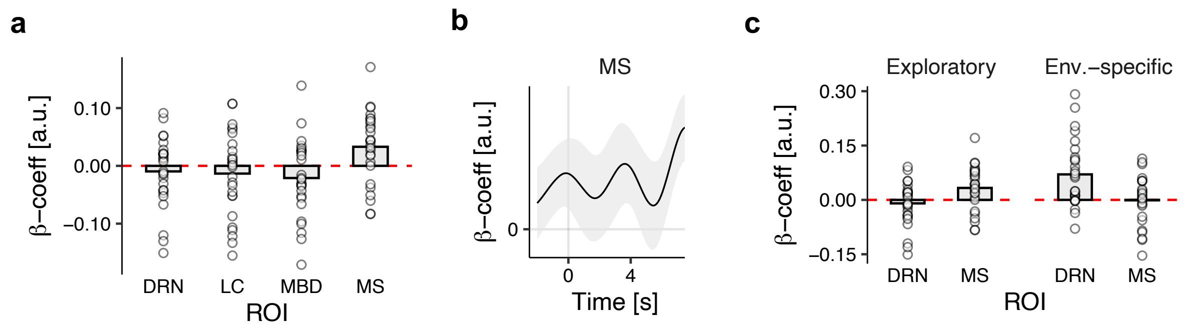


**Figure S7. Analysis of exploratory policy switches. (a–b)** Relationship between activity in subcortical ROIs and incongruent policy-switches – i.e., pursue-to-reject switches in poor environments and reject-to-pursue switches in rich environments. Activity in MS, but no other subcortical ROI, was modulated by these events (GLM S7.1; $t_{MS;\ incgongruent\text{-}switch}(26)=2.80$ , $p$=.047). This is broadly consistent with the link between cholinergic pathways and explorative forms of behaviour, in the sense that incongruent policy switches are deviations from an established reward-maximising strategy. Line and shading indicates mean ± SE of regression-weight across participants. Bars indicate sample-level mean peak regression weights; dots indicate participant-level peak regression weights. **(c)** Effect of exploratory and environment-driven policy switches in DRN and MS activity. An ANOVA indicated an ROI-by-switch-typed (GLM S7.2; $ANOVA_{ROI*switch\text{-}type} = 10.95$, p = .001), whereby environment-driven switch-signals were stronger in DRN than MS ($t_{DRN\text{-}vs\text{-}MS;\ environment\text{-}specific}(26) = 3.61$, $p$=.001). This indicates that DRN activity – but not MS activity – represented policy-switches linked to the richness of the environment, whereas MS activity – but not DRN activity – represented policy-switches that were incongruent with the environment. Bars indicate sample-level mean peak regression weights; dots indicate participant-level peak regression weights. In all panels, n=27.

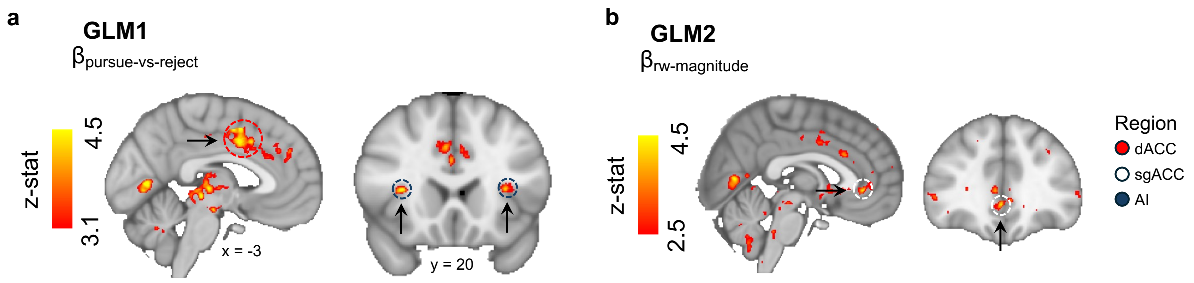


**Figure S8. Whole-brain fMRI analysis for identifying regions of interest for representational similarity analysis.** Regions of interest (ROIs) for representational similarity analysis (RSA) were identified with two whole-brain GLMs. **(a)** The first (GLM3.1) concerned activity related to the pursue-vs-reject decision made on each trial. Clusters from GLM3.1 in dorsal anterior cingulate cortex (dACC) centred on the cingulate sulcus and extending dorsally into supplementary and presupplementary motor areas (*z*-max = 4.95; MNI-coordinates = [-1, 5, 38]). Additional clusters from GLM3.1 in anterior insula cortex (AI) at the border with frontal operculum (left z-max = 3.89, MNI-coordinates = [-32, 10, 10]; right z-max = 4.36, MNI-coordinates = [43, 9, 3]). **(b)** The second (GLM3.2) concerned activity related to the factors influencing pursue-vs-reject decisions. Clusters from GLM3.2 in subgenual anterior cingulate cortex (sgACC) related to the reward-magnitude of the opportunity on each trial (*z*-max=3.87, MNI coordinates = [2, 36, -3]). Although the z-score for this cluster is below the conventional cluster-correction threshold of *z*=3.1, we retained it as an ROI because sgACC and neighbouring areas like perigenual anterior cingulate cortex (pgACC) have been linked neural representations subjective value, which is in principle similar to the relative-value computations we investigated here.

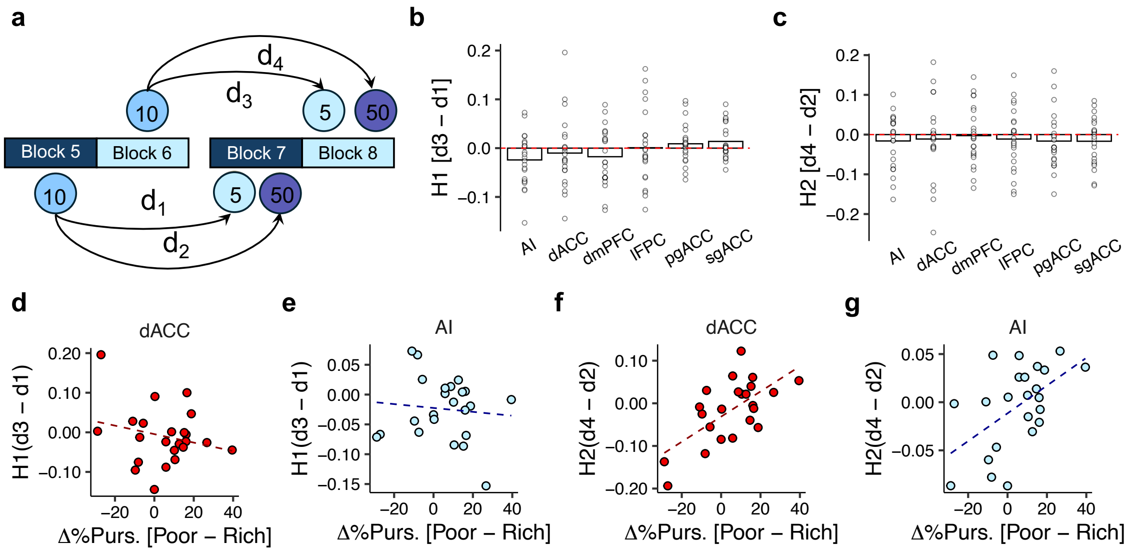


**Figure S9. Further detail of representational similarity analysis. (a)** Diagram of distance calculation logic during representational similarity analysis (RSA), which involved comparing pairwise representational distances between reward options in non-contiguous rich and poor blocks to avoid similarity artefacts from autocorrelations in BOLD signal. **(b)** H1 – the representational distance between 10-point and 5-point options in poor compared to rich blocks – in all cortical ROIs. There was no evidence that H1 was different to 0 in any ROI. Bars indicate sample-level mean H1 values; dots indicate participant-level H1 values. **(c)** H2 – the representational distance between 10-point and 50-point options in poor compared to rich blocks – in all cortical ROIs. There was no evidence that H2 was different to 0 in any ROI. There was no evidence that representations of 10-point and 50-point options were different between poor and rich blocks. Bars indicate sample-level mean H2 values; dots indicate participant-level H2 values. **(d)** Relationship between H1 – the representational distance between 10-point and 5-point options in poor compared to rich blocks – and behaviour in dACC. There was no evidence of correlation between H1 and behaviour ($t_{dACC}(21)$ = -1.12, *p*=.271) Dots indicate observations from individual participants, line indicates line-of-best-fit. **(e)** Relationship between H1 and behaviour in AI. There was no evidence of correlation between H1 and behaviour ($t_{AI}(21)$ = -0.45, *p*=.650). Dots indicate observations from individual participants, lines indicates line-of-best-fit. **(f–g)** We verified that the RSA results in the main text are not an artefact of computing representational distance with cosine similarity by replicating the analysis suggests Pearson's correlation ($r_{ACC}$=0.65, $t(21)$= 3.91, $p$<.001; $r_{AI}$=0.53, $t(21)$=2.90, $p$=.008). This suggest that the result obtains regardless of the representational distance metric employed. In all panels, n=27.

## SUPPLEMENTARY TABLES

### Table 1: fMRI cluster locations for GLM3.1

Clusters for $\beta_3$decision(opportunity-onset) contrast in GLM 3.1

| Anatomical location | x | y | z | *z*-max | *p* | Size (# voxels) |
|---|---|---|---|---|---|---|
| Left Thalamus | -15 | -21 | 7 | 5.28 | 9.61e-32 | 7132 |
| Postcentral Gyrus | -56 | -18 | 44 | 6.18 | 9.54e-31 | 6811 |
| Anterior Cingulate Gyrus | -1 | 5 | 38 | 4.95 | 1.6e-30 | 6739 |
| Right Cerebellum | 18 | -50 | -20 | 6.04 | 2.03e-29 | 6391 |
| Occipital Pole | 16 | -95 | 4 | 5.15 | 1.46e-19 | 3570 |
| Insular/Central Opperculum | -39 | -4 | 16 | 5.65 | 6.35e-16 | 2675 |
| Right Cerebellum | 17 | -58 | -46 | 4.72 | 1.58e-14 | 2355 |
| Left Cerebellum | -23 | -59 | -19 | 4.63 | 3.42e-11 | 1648 |
| Precentral Gyrus | -55 | 6 | 36 | 5.12 | 5.71e-11 | 1604 |
| Lingual Gyrus | -3 | -71 | 4 | 4.79 | 5.96e-08 | 1068 |
| Posterior Cingulate Gyrus/Precentral Gyrus | -8 | -24 | 44 | 5.16 | 8.34e-07 | 855 |
| Left Putamen | -32 | -8 | -2 | 4.44 | 7.45e-06 | 709 |
| Occipital Pole/Superior Lateral Occipital Cortex | -22 | -93 | 9 | 4.65 | 1.08e-05 | 685 |
| Inferior Lateral Occipital Cortex | -41 | -85 | 9 | 4.54 | 0.000365 | 471 |
| Lateral Frontal Pole (Right) | 30 | 59 | 0 | 4.14 | 0.000528 | 450 |
| Postcentral Gyrus/Anterior Supramarginal Gyrus | 40 | -31 | 41 | 4.31 | 0.00144 | 395 |
| Lateral Frontal Pole (Left) | -24 | 63 | 2 | 3.89 | 0.00155 | 391 |
| Insular/Central Opperculum (Right) | 44 | 2 | 11 | 3.91 | 0.00289 | 358 |
| Anterior Insular/Frontal Opperculum (Left) | -32 | 19 | 10 | 4.29 | 0.00461 | 334 |
| Cerebral White Matter (Right) | 24 | -23 | -4 | 4.56 | 0.00789 | 307 |
| Posterior Cingulate Gyrus/Precentral Gyrus (Right) | 16 | -31 | 39 | 4.01 | 0.0269 | 248 |
| Central Opperculum/Insular | 43 | 9 | 3 | 4.36 | 0.0334 | 238 |
| Anterior Insular/ Frontal Opperculum/ | 33 | 21 | 9 | 4.69 | 0.0484 | 221 |

**Table 2: fMRI cluster locations for GLM3.2**

Clusters for $\beta_3$reward-magnitude contrast in GLM3.2

| Anatomical location | x | y | z | *z*-max | *p* | Size (# voxels) |
|---|---|---|---|---|---|---|
| Right Cerebellum | 20 | -50 | -20 | 4.35 | 9.48e-20 | 197 |
| Occipital Pole | 15 | -92 | 3 | 4.6 | 4.9e-15 | 135 |
| Postcentral Gyrus | -56 | -19 | 45 | 4.28 | 6.16e-13 | 110 |
| Insular/Central Opercular Cortex | -40 | -3 | 15 | 4.41 | 1.42e-10 | 84 |
| Postcentral/Supramarginal Gurys | -58 | -23 | 23 | 3.98 | 1.19e-07 | 55 |
| Lingual Gyrus | -4 | -74 | -2 | 4.15 | 1.31e-06 | 46 |
| Anterior Cingulate Cortex/Cingulate Gyrus | - 6 | 6 | 43 | 4.05 | 5.13e-06 | 41 |
| Right Nucleus Accumbens | 6 | 11 | -5 | 4.71 | 2.12e-05 | 36 |
| Supramarginal Gyrus | -59 | -34 | 31 | 4.01 | 2.83e-05 | 35 |
