# Supplementary material for: Activity in human dorsal raphe nucleus signals changes in behavioural policy
Source: Nat Commun. 2026 Feb 14;17:1665. doi: 10.1038/s41467-026-68349-9 (PMC12909899; doi:10.1038/s41467-026-68349-9)
Supplement: Supplementary file 2 — Reporting Summary [file 41467_2026_68349_MOESM2_ESM.pdf]

nature portfolio

Corresponding author(s): Luke Priestley
Last updated by author(s): 25-08-18

Reporting Summary

Nature Portfolio wishes to improve the reproducibility of the work that we publish. This form provides structure for consistency and transparency in reporting. For further information on Nature Portfolio policies, see our Editorial Policies and the Editorial Policy Checklist.

Statistics

For all statistical analyses, confirm that the following items are present in the figure legend, table legend, main text, or Methods section.

n/a	Confirmed	
☐	☒	The exact sample size (*n*) for each experimental group/condition, given as a discrete number and unit of measurement
☐	☒	A statement on whether measurements were taken from distinct samples or whether the same sample was measured repeatedly
☐	☒	The statistical test(s) used AND whether they are one- or two-sided<br>*Only common tests should be described solely by name; describe more complex techniques in the Methods section.*
☐	☒	A description of all covariates tested
☐	☒	A description of any assumptions or corrections, such as tests of normality and adjustment for multiple comparisons
☐	☒	A full description of the statistical parameters including central tendency (e.g. means) or other basic estimates (e.g. regression coefficient) AND variation (e.g. standard deviation) or associated estimates of uncertainty (e.g. confidence intervals)
☐	☒	For null hypothesis testing, the test statistic (e.g. *F*, *t*, *r*) with confidence intervals, effect sizes, degrees of freedom and *P* value noted<br>*Give P values as exact values whenever suitable.*
☒	☐	For Bayesian analysis, information on the choice of priors and Markov chain Monte Carlo settings
☐	☒	For hierarchical and complex designs, identification of the appropriate level for tests and full reporting of outcomes
☒	☐	Estimates of effect sizes (e.g. Cohen's *d*, Pearson's *r*), indicating how they were calculated

Our web collection on statistics for biologists contains articles on many of the points above.

Software and code

Policy information about availability of computer code

Data collection	The experiment was written in MATLAB R2022a (Mathworks, USA) using the Psychophysics Toolbox extension.
Data analysis	Data analysis was performed in MATLAB R2022a (Mathworks, USA), and R version 4.2.2. Mixed-effect modelling was conducted using l'me4' and 'optimx' packages in R. Pre-processing and analysis of fMRI data was performed using tools from FMRIB's Software Library (FSL). Custom scripts for data analysis are available at: https://github.com/lpriestley/nhb_materials

For manuscripts utilizing custom algorithms or software that are central to the research but not yet described in published literature, software must be made available to editors and reviewers. We strongly encourage code deposition in a community repository (e.g. GitHub). See the Nature Portfolio guidelines for submitting code & software for further information.

Data

Policy information about availability of data

All manuscripts must include a data availability statement. This statement should provide the following information, where applicable:

- Accession codes, unique identifiers, or web links for publicly available datasets
- A description of any restrictions on data availability
- For clinical datasets or third party data, please ensure that the statement adheres to our policy

Anonymised behavioural and fMRI data used in the manuscript are available at: https://github.com/lpriestley/nhb_materials

Research involving human participants, their data, or biological material

Policy information about studies with human participants or human data. See also policy information about sex, gender (identity/presentation), and sexual orientation and race, ethnicity and racism.

Reporting on sex and gender	The study included participants who self-reported as being women (N=20) or men (N=11)
Reporting on race, ethnicity, or other socially relevant groupings	Information not collected.
Population characteristics	Information not collected
Recruitment	All participants were between 18-40 years of age, reported normal or corrected-to-normal vision, and no current diagnosis or treatment for psychiatric or neurological disorder. Participants received £40 for completing the experiment and could earn an additional payment of up to £10 depending on their performance in the decision-making task.
Ethics oversight	Oxford University Central University Research Ethics Committee (CUREC)

Note that full information on the approval of the study protocol must also be provided in the manuscript.

Field-specific reporting

Please select the one below that is the best fit for your research. If you are not sure, read the appropriate sections before making your selection.

☐ Life sciences ☒ Behavioural & social sciences ☐ Ecological, evolutionary & environmental sciences

For a reference copy of the document with all sections, see nature.com/documents/nr-reporting-summary-flat.pdf

Behavioural & social sciences study design

All studies must disclose on these points even when the disclosure is negative.

Study description	Quantitative experimental data.
Research sample	Twenty-nine participants performed the experiment. All were between 18-40 years of age, reported normal or corrected-to-normal vision, and no current diagnosis or treatment for psychiatric or neurological disorder. Participants included students, university staff, and members of the general public from Oxford, UK. The participants, therefore, were representative of a university town in the UK. All analyses focused on behavioural and neural data collected from all participants, and there was no attempt to define or compare different sub-groups within the sample.
Sampling strategy	Random sampling was used. The sample size was based on previous ultra-high field neuroimaging studies with similar behavioural paradigms (Khalighinejad et al., 2020, PNAS; Khalighinejad et al., 2022, Nature Communications).
Data collection	Participants performed the experiment on a laptop computer running MATLAB R2022a (Mathworks, USA) and in a 7T MRI scanner (Siemens). The researcher was not blind to the experimental conditions, nor to the hypotheses motivating the study.
Timing	Data collection took place from May 2022 to May 2023.
Data exclusions	Two participants were exlcuded because they did not perform the task correctly. Two additional participants were excluded because of excessive head motion, which prevented accurate registration during fMRI analysis.
Non-participation	All participants completed the study.
Randomization	The study had a within-subjects design, and therefore no group allocation was necessary.

Reporting for specific materials, systems and methods

We require information from authors about some types of materials, experimental systems and methods used in many studies. Here, indicate whether each material, system or method listed is relevant to your study. If you are not sure if a list item applies to your research, read the appropriate section before selecting a response.

Materials & experimental systems

n/a	Involved in the study
☒	☐ Antibodies
☒	☐ Eukaryotic cell lines
☒	☐ Palaeontology and archaeology
☒	☐ Animals and other organisms
☒	☐ Clinical data
☒	☐ Dual use research of concern
☒	☐ Plants

Methods

n/a	Involved in the study
☒	☐ ChIP-seq
☒	☐ Flow cytometry
☐	☒ MRI-based neuroimaging

Plants

Seed stocks	*Report on the source of all seed stocks or other plant material used. If applicable, state the seed stock centre and catalogue number. If plant specimens were collected from the field, describe the collection location, date and sampling procedures.*
Novel plant genotypes	*Describe the methods by which all novel plant genotypes were produced. This includes those generated by transgenic approaches, gene editing, chemical/radiation-based mutagenesis and hybridization. For transgenic lines, describe the transformation method, the number of independent lines analyzed and the generation upon which experiments were performed. For gene-edited lines, describe the editor used, the endogenous sequence targeted for editing, the targeting guide RNA sequence (if applicable) and how the editor was applied.*
Authentication	*Describe any authentication procedures for each seed stock used or novel genotype generated. Describe any experiments used to assess the effect of a mutation and, where applicable, how potential secondary effects (e.g. second site T-DNA insertions, mosiacism, off-target gene editing) were examined.*

Magnetic resonance imaging

Experimental design

Design type	Event-related
Design specifications	The task consisted in 10 4.5 min blocks of 15-20 trials. The precise number of trials in each block varied depending on how frequently participants pursued or rejected reward opportunities because pursuing a reward opportunity was accompanied by a time-cost. Each trial began with an inter-trial-interval (ITI; ITI ~TruncExp(μ = 4.5 , min=3.5, max=5.5). Participants needed to consider each reward-option for a short duration (Opportunity ~TruncExp(μ = 4.5 , min=3.5, max=5.5) before receiving a go-cue. Participants could pursue options by making a button press response within 1s of the go-cue. Pursuing an opportunity incurred a temporal opportunity cost via action-outcome (Action-outcome ~ TruncExp(μ = 4.5 , min=3.5, max=5.5) and reward feedback delays (Rw-feedback ~ TruncExp(μ = 4.5 , min=3.5, max=5.5). The duration of these delays was equivalent to the time taken by one future encounter – in other words, pursuing an opportunity on the current trial meant foregoing a reward opportunity in the future.
Behavioral performance measures	Button-presses and response times were recorded. Behavioural was modelled using generalised mixed-effects models with by-subject random intercepts and by-subject random slopes, as implemented in the lme4 package in R. We verified that participants understood the task by examining how frequently they pursued each option, with the expectation that high-value options (e.g. the 50-point) would be pursued more or as frequently as low value options (5-point).

Acquisition

Imaging type(s): Functional and structural

Field strength: 7T

Sequence & imaging parameters:

Structural and functional MRI data was collected with a Siemens 7 Tesla MRI scanner. High-resolution functional data were acquired with a multiband gradient-echo T2* echo planar imaging sequence with 1.5mm isotropic voxels, multiband acceleration factor 2, repetition time (TR) = 1.775s, echo time (TE) = 17.8ms, flip angle = 66°, and GRAPPA acceleration factor 2. The parameters were selected to maximise signal-to-noise ratio in subcortical areas. To accommodate the high temporal and spatial resolution of the protocol, functional scans had a limited field of view (FOV) oriented at 30 degrees with respect to the AC-PC line (66 slices with a coverage of 99 mm). The FOV captured all regions of interest in the midbrain, brainstem and cortex. Before acquiring the task-related functional scan, we acquired a pre-saturation single-measurement, whole-brain functional scan with the same orientation. The pre-saturation scan was used to facilitate registration of the limited-FOV task-related functional scan to the whole brain.

Structural data were acquired using a T1-wieghted MP-RAGE sequence with 0.7mm isotropic voxels, GRAPPA acceleration factor 2, TR = 2200ms, TE = 3.02ms, and; inversion time (TI) = 1050ms. To correct distortions arising from inhomogeneities in the magnetic field, a fieldmap sequence was acquired with 2mm isotropic voxels, TR = 620ms, TE1 = 4.08ms, and TE2 = 5.1ms. To account for the effects of physiological noise on functional MRI data, participants were fitted with a pulse oximeter and respiratory bellows that acquired cardiac and respiratory timeseries at 50Hz using a BioPac MP160 device (BIOPAC Systems Inc., USA).

Area of acquisition: Partial acquisition chosen to cover brainstem, modbrain and frontal cortex.

Diffusion MRI: ☐ Used ☒ Not used

Preprocessing

Preprocessing software:

Preprocessing of fMRI data was performed with the FMRIB Software Library (Jenkinson et al., 2012; Smith et al., 2004). The Brain Extraction Tool (Smith, 2002) was used to separate brain from non-brain matter in structural and functional images. Functional images were normalised, spatially smoothed (Gaussian kernel with a 3mm full-width half-maximum) and temporally high-pass filtered (3 dB cut-off = 100s), and artefacts arising from head motion were removed using MCFLIRT (Jenkinson et al., 2002).

Normalization:

Registration of task-related functional images to Montreal Neurological Institute (MNI)-space was performed in three stages:
1. The task-related limited-FOV EPI was registered to the pre-saturation whole-brain EPI using FMRIB's Linear Image Registration Tool with 6 degrees of freedom transformation.
2. The whole-brain EPI was registered to the subject-specific structural images using Boundary-Based Registration (BBR) incorporating fieldmap correction (Greve & Fischl, 2009).
3. Subject-specific structural images were registered to a 1mm resolution Standard MNI template with FMRIB's Non-linear Registration Tool (FNIRT; (Jenkinson et al., 2012)).

Normalization template: MNI152

Noise and artifact removal:

Non-task confound regressors were added to reduce noise in BOLD signal, including: (1) head motion parameters estimated using MCFLIRT during pre-processing (Jenkinson et al., 2002); (2) regressors for voxel-wise estimates of physiological noise arising from cardiac and respiratory activity, estimated using FSL's Physiological Noise Monitoring (PNM) tool (Brooks et al., 2008), and; (3) regressors for motion outliers, indicating volumes with head motion that could not be corrected with linear methods.

Volume censoring:

FSL's Motion Outlier tool was used to identify volumes corrupted by large movements. The Motion outliers tool creates confound matrix for used in GLMs which completely removes the effects of identified volumes on analysis without adverse effects on the statistics. On average, 5.98% of volumes were identified as corrupted due to large head motions.

Statistical modeling & inference

Model type and settings:

Statistical analysis of whole-brain functional data was performed at two levels using FMRIB's Expert Analysis Tool (FEAT; (Jenkinson et al., 2012)). In the first level, a univariate general linear model was used to compute parameter estimates for each regressor in each participant (Woolrich et al., 2001). Contrast and variance estimates for each parameter in each participant were subsequently combined in a mixed-effects analysis conducted at the second level (FLAME 1 + 2), where subject-identity was a random effect (Woolrich et al., 2004).

For time series analysis on ROI data, the filtered time-series of BOLD signal from each voxel was averaged, normalised and up-sampled by a factor of 20 with spline interpolation. Up-sampled timeseries were then epoched in 10s windows starting 2s before and ending 8s after reward-option onset on each trial. Timeseries data was analysed by fitting a GLM with ordinary least squares (OLS) at each timepoint in each epoch.

Effect(s) tested:

All models used to test effects of interest are described in the Methods section. For whole-brain analyses, see GLMs 3.1–3.2. For ROI analyses, see GLMs 4.1–4.2. For RSA, see GLM 5.1. Significance testing for timecourse ROI GLMs was assessed using a leave-one-out procedure that avoided temporal biases in the selection of peak-effects

Specify type of analysis: ☐ Whole brain ☐ ROI-based ☒ Both

Anatomical location(s)

Anatomical ROIs were constructed for a series of brainstem and midbrain nuclei previously implicated in reward-guided decision-making including dorsal raphe nucleus (DRN), Locus Coeruleus (LC), Medial Septum (MS), a combined mask covering the Substantia Nigra and Ventral Tegmental Area (Midbrain Dopaminergic Nuclei; MDB), and Habenula (Hb). The LC mask derived from a brain atlas developed by Pauli and colleagues (Pauli et al., 2018). DRN, MDB, Hb and MS masks were manually drawn in MNI-space based on an atlas of the human brain atlas (Mai et al., 2016). The DRN mask was drawn in consultation with the anatomical guidelines of Kranz and colleagues (Kranz et al., 2012) to ensure specificity to the dorsal portion of raphe nucleus. To ensure conformation between anatomical masks and subject-specific neuroanatomy, subject-specific masks were manually checked and edited by two experimenters and evaluated for inter-rater reliability by a third experimenter. Structural-space masks were then transformed from structural to functional space by applying a structural-to-functional affine matrix, and were subsequently thresholded, binarized and dilated by one voxel.

Statistic type for inference

(See Eklund et al. 2016)

For whole brain analyses, results were cluster-corrected with a voxel inclusion threshold of X=3.1 and cluster significance threshold of p=.001. Data where pre-whitened before analysis to account for temporal autocorrelations in BOLD signal. For time-series analysis of ROI data, we used a leave-one out cross validation procedure described above.

Correction

For whole-brain GLMs, FWE was used with p<.001. For ROI analyses, Bonferroni correction was used.

Models & analysis

n/a	Involved in the study
☐	☒ Functional and/or effective connectivity
☒	☐ Graph analysis
☐	☒ Multivariate modeling or predictive analysis

Functional and/or effective connectivity

Psychophysiological interaction analysis was used to model interactions between DRN and AI/dACC as a function of the richness of the environment.

Multivariate modeling and predictive analysis

Multivariate pattern analysis (MVPA) was used to model responses evoked by each reward option (5-point, 10-point, 50-point) in each kind of block-type (rich-blocks vs poor-blocks). The full details of this analysis are described in the Methods section.
